# Injuries from Electricity

**Published:** 1889-12-14

**Authors:** 


### SURGERY.

### INJURIES FROM ELECTRICITY.

We have heard a good deal latterly about injuries from electricity, the newspapers having recently recorded several such accidents. The subject has been discussed by Dr. Charles L. Dana in the New York *Medical Record* of November 2. The means by which the electrical current does harm varies with the form in which it is used. The telegraph and telephone produce peculiar neuroses, due not to the electrical current, but to the peculiar demand made upon the nervous system of the operator, the results being telegraphists' cramp, aural and mental disturbances, etc. Most of the other observed injuries from electricity arise from the apparatus carrying electrical currents for lighting and power. Such currents, when received into the human system, produce varying effects in accordance with the mode of contact and the susceptibility of the individual. In some cases they merely stun the victim, and burn the parts in contact with the wire. In others, they produce permanent paralysis. In still other instances instantaneous death results. But sometimes a mental shock is produced, which affects the system much as other shocks do, causing traumatic hysteria or neurasthenia. The electrical current burns, or not, according to the dryness of the skin and clothes. With a dry skin, there is more burning and less shock; with a wet skin, the reverse. It has been noted that the most fatal electrical accidents have occurred on rainy days. When a person, by accident, receives a strong electrical current, "it promptly knocks the recipient down," and may cause a temporary tetanus—even if the current is at once broken. Consciousness is not usually lost, but severe pain and terror are felt. In a few minutes the muscles relax, and the patient is able to move. The arm or leg—or whatever part was in contact with the wire—feels numb for hours or longer. Dr. Dana states that electricity, even in fatal quantities, does not usually much affect the nervous structure, but tends rather to cause carbonisation of the blood and distension of vessels, and sometimes hæmorrhages, with occasional disruption of muscular and vascular or other tissue. Cases are detailed illustrating Dr. Dana's views. The extraordinary increase now going on in the practical application of electricity has certainly resulted in the production of a new class of injuries, injuries which have heretofore been produced by lightning only, and which have, at least in this country, been comparatively rare. From some statistics which Dr. Dana gives it appears that the use of electricity for various purposes prevails to a greater extent in America than at present in this country. It is probably this fact which has led the author of the very interesting paper we have briefly summarised to take up the subject. But judging from what is going on here in the extension of electric lighting and power, it will soon be necessary for us to follow Dr. Dana's example. Then the monograph referred to will be found most useful and suggestive as the basis of further investigation.

